# Global consumption patterns of combination hypertension medication: An analysis of pharmaceutical sales data from 2010–2021

**DOI:** 10.1371/journal.pgph.0003698

**Published:** 2024-09-06

**Authors:** Sahan Jayawardana, Allen Campbell, Murray Aitken, Charlotte E. Andersson, Mandeep R. Mehra, Elias Mossialos

**Affiliations:** 1 Department of Health Policy, LSE Health, London School of Economics and Political Science, London, United Kingdom; 2 IQVIA Institute for Human Data Science, Parsippany, New Jersey, United States of America; 3 Department of Medicine, Brigham and Women’s Hospital and Harvard Medical School, Boston, Massachusetts, United States of America; Weill Cornell Medicine - Qatar, QATAR

## Abstract

Hypertension is the most significant risk factor for cardiovascular disease and mortality worldwide, affecting 1.3 billion adults. Global disparities in hypertension control are widening with low- and middle-income countries (LMIC) having the fastest growing rates of hypertension and low rates of control. Treatment for hypertension can be challenging, with multiple drug classes and dosing schedules. Combination antihypertensives have been suggested as a solution for their efficacy and potential to improve adherence. Global consumption of combination and non-combination antihypertensives across 75 countries and 2 regions from 2010 to 2021 was estimated using the IQVIA MIDAS database on pharmaceutical sales. Consumption rates were standardized using Standard Units (SUs) and analysed by high-income (HIC), upper-middle income (UMIC), and LMIC income classification. Global median consumption rate of all antihypertensives per 1000 inhabitants per day increased from 184.78 SUs in 2010 to 325.6 SUs in 2021, with HICs consistently having the highest rates. Median consumption rates of combination and non-combination antihypertensives increased across all country income groups but combination drugs were consumed at a lower rate and proportion. LMICs consumed a higher percentage of combination antihypertensives relative to non-combination (45.5%) than UMICs (24.3%) and HICs (24.4%) in 2021. While combination antihypertensives may be preferred for their potential for increased adherence and effectiveness, their global uptake is inconsistent. HICs consume less combination medication relative to non-combination, despite higher overall consumption rates of antihypertensives. LMICs show increasing use of combination medications, indicating a shift towards their use.

## Introduction

Hypertension is the most significant risk factor for cardiovascular disease and mortality worldwide, affecting 1.3 billion adults [1]. Despite approximately 30% of the global population suffering from hypertension, only 13.8% of these cases were managed effectively [2–4]. Uncontrolled hypertension escalates the risk for overt cardiovascular and kidney disease, and was responsible for 10.8 million deaths in 2019, predominantly through its attribution to ischemic heart disease and cerebrovascular disease [5, 6]. Additionally, hypertension prevalence and control show significant global disparities. Over the last two decades, hypertension rates have declined in high-income countries but increased in low- and middle-income countries (LMIC), with over 1 billion of the total hypertension patients in 2019 (82% of total population with hypertension) residing in LMICs where insufficient access to drugs contributes to low treatment rates [1].

While pharmacologic agents, such as anti-hypertensive medications, are key for long-term control, the vast range of available drug classes, doses, and potencies complicate treatment regimens. Most patients require concurrent treatment with multiple antihypertensive medications for effective control, making adherence difficult [7–9]. One potential solution is combination antihypertensive medications, which merge two or more hypertension drug classes into a single pill [9]. These medications not only simplify the treatment regimen but also provide a stronger blood pressure reduction effect than either medication alone [10, 11]. These combination pills lower the required dosage of each agent, reducing adverse medication-related side effects and improving adherence rates [10–12].

The evidence base for combination antihypertensives was established largely in high-income countries, which were the first to include them in clinical guidelines [10, 12, 13]. There is limited evidence on the use of combination antihypertensives in middle- or lower-income countries. To the best of our knowledge, no comprehensive study has previously assessed the consumption patterns of combination antihypertensives from a global perspective. Given previous pharmaceutical consumption patterns in wealthier nations [14, 15], our hypothesis is that high-income countries would have both higher overall rates of antihypertensive medications and a larger percentage of combination medication use in comparison to their total antihypertensive medication consumption.

To evaluate our hypothesis, we analysed global sales data of combination and non-combination antihypertensive medicines in 75 countries and 2 regions, representing more than 80% of the global population, from 2010 to 2021. We calculated the antihypertensive consumption rates for each country and evaluated the consumption patterns of combination vs single medication formulations of antihypertensive drugs by country income classification.

## Methods

To assess consumption of antihypertensive drugs, we utilized the IQVIA MIDAS database, a resource previously employed for assessing global antibiotic and opioid usage [14]. IQVIA collects data on antihypertensives using the same approach. The data in the IQVIA MIDAS database originates from the supply chain at the national level, primarily through shipments to delivery points such as pharmacies, hospitals, and clinics from manufacturers, distributors, and wholesalers. Sometimes, data is gathered as consumption based on distribution to patients. The exact data collection point can differ according to the healthcare system and distribution model of each country. Most data is electronically collected and reported on a monthly or quarterly basis. We used quarterly sales data for both hospital and retail settings from 2010 to 2021 across 75 countries and 2 additional regions. Countries included in the 2 regions were: Central America (Costa Rica, El Salvador, Guatemala, Honduras, Nicaragua, and Panama) and French-speaking west Africa (Benin, Burkina Faso, Cameroon, Chad, Côte d’Ivoire, Republic of Congo, Guinea, Mali, Niger, Senegal, and Togo).

We had complete data for 50 countries or regions. For 27 countries, we had complete sales data for the retail sector but no data from the hospital sector of these countries. However, within these 27 countries, the retail sector accounted for more than 70% of the antihypertensives market in 20 countries and 60% or more in 3 countries. Therefore, the missing data for the hospital sector market in these countries is unlikely to substantially impact our consumption rate estimates, trends and key conclusions. We report the retail market share for these 27 countries in S1 Table.

Antihypertensive drugs were included based on the following European Pharmaceutical Marketing Research Association (EphMRA) Anatomical Therapeutic Chemical 2022 classification codes: C2 (antihypertensives), C2A (antihypertensives of non-herbal origin, plain), C2B (antihypertensives of non-herbal origin, combination with diuretics), C2C Rauwolfia alkaloids and other antihypertensives of herbal origin, C2D Rauwolfia alkaloids and other antihypertensives of herbal origin in combination with diuretics, C3 Diuretics, C7 beta blockers, C8 calcium antagonists with antihypertensive and/or anti-angina and C9 ACE inhibitors and ATII inhibitors.

Each antihypertensive drug has unique dosing methods and potency levels. To standardise potency and dosing across all medications, IQVIA translates volume information into Standard Units (SUs), a measure of volume created by IQVIA to enable comparison between countries. The Standard Unit for any medication corresponds to the single-dose unit for that drug, which could be a single capsule, pill, or a comparable quantity of liquid. The calculation of SUs depends on the form of the medication and is based on the principle of converting the medication quantity into a standard measurement. For example, one standard unit for solid dosage forms equals one tablet or capsule while for liquid dosage forms, an SU may be defined as a milliliter (ml) or another volume measure that corresponds to a typical dose. The total volume dispensed is converted into Standard Units based on this measure. Previous studies have used SUs to standardize dosing across medications and to facilitate comparisons among different medications [15, 16]. We assumed antihypertension drug consumption as the volume of sales expressed in SUs. The annual consumption rate of antihypertension drugs was expressed as SUs per 1000 individuals, using population estimates from the World Bank [17]. Countries were grouped by income class as high (HIC), upper-middle (UMIC), and low and lower-middle (LMIC) using the 2016 World Bank income classification.

We assessed the variations in antihypertension drug usage based on two factors: 1) the change in the annual consumption rate for each country from 2010 to 2021; and 2) the differences in the median annual consumption rate trends among the three income groups over the course of the study period. We assessed the differences in trends using the Wald chi-square test. Additionally, we examined the differences in the usage of combination and non-combination antihypertensive medications across income groups as a percentage of total antihypertensive medications consumed. We also report these results for the year 2019 to assess sensitivity to the potential impact of the COVID-19 pandemic in 2020/21.

Statistical analyses were performed using R statistical software, version 4.3.1 (R Foundation).

## Results

**Fig 1:**
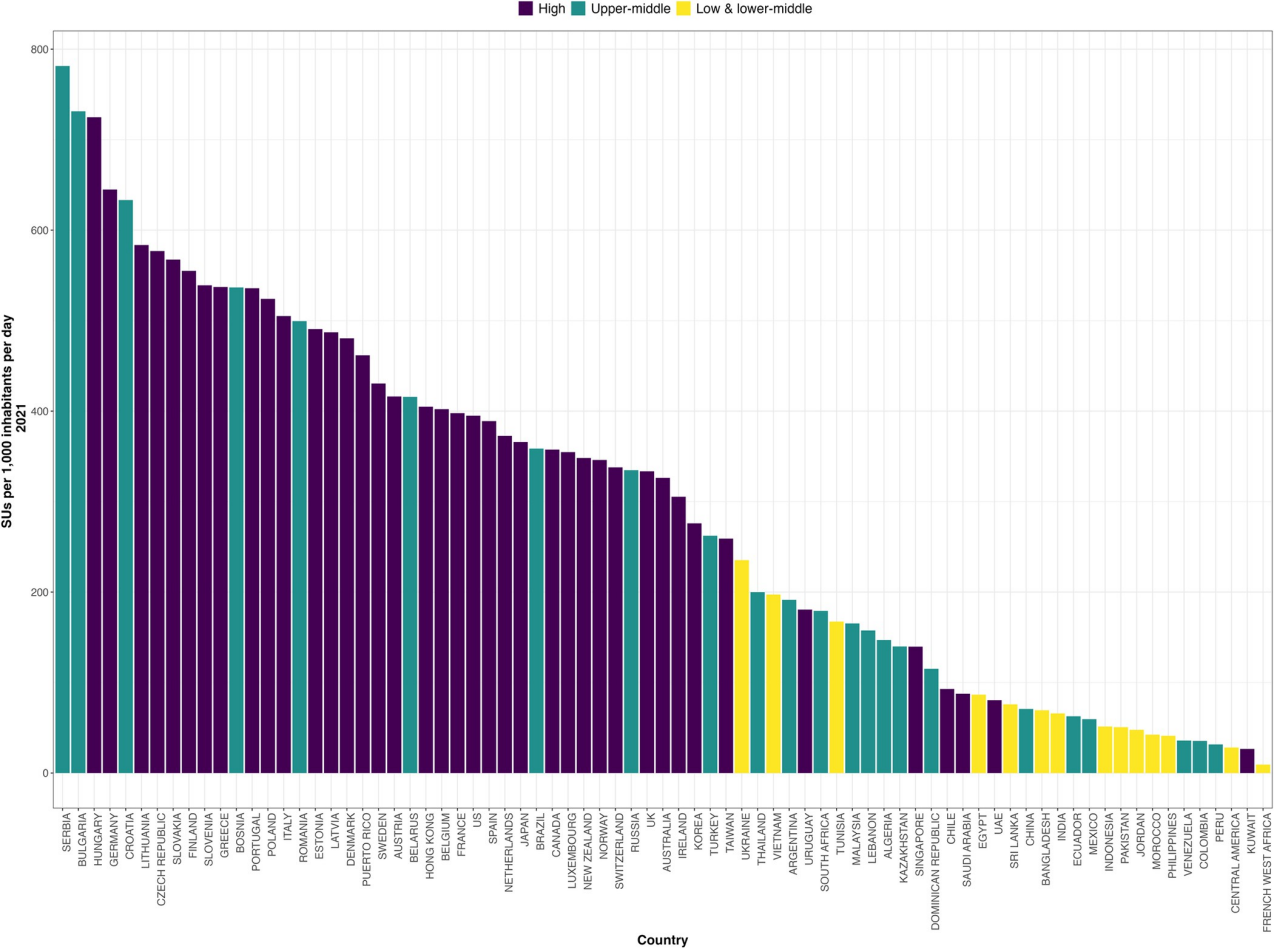
SU consumption rate by country for 2021 in SUs per 1000 inhabitants per day.

In 2021, Serbia, Bulgaria, and Hungary had the highest SUs consumed per 1,000 inhabitants per day (Fig 1). Between 2010 and 2021, Croatia (48.45 SUs), Austria (13.74 SUs), and Lithuania (10.94 SUs) had the greatest increases in the national antihypertensive medication consumption rate.

**Fig 2:**
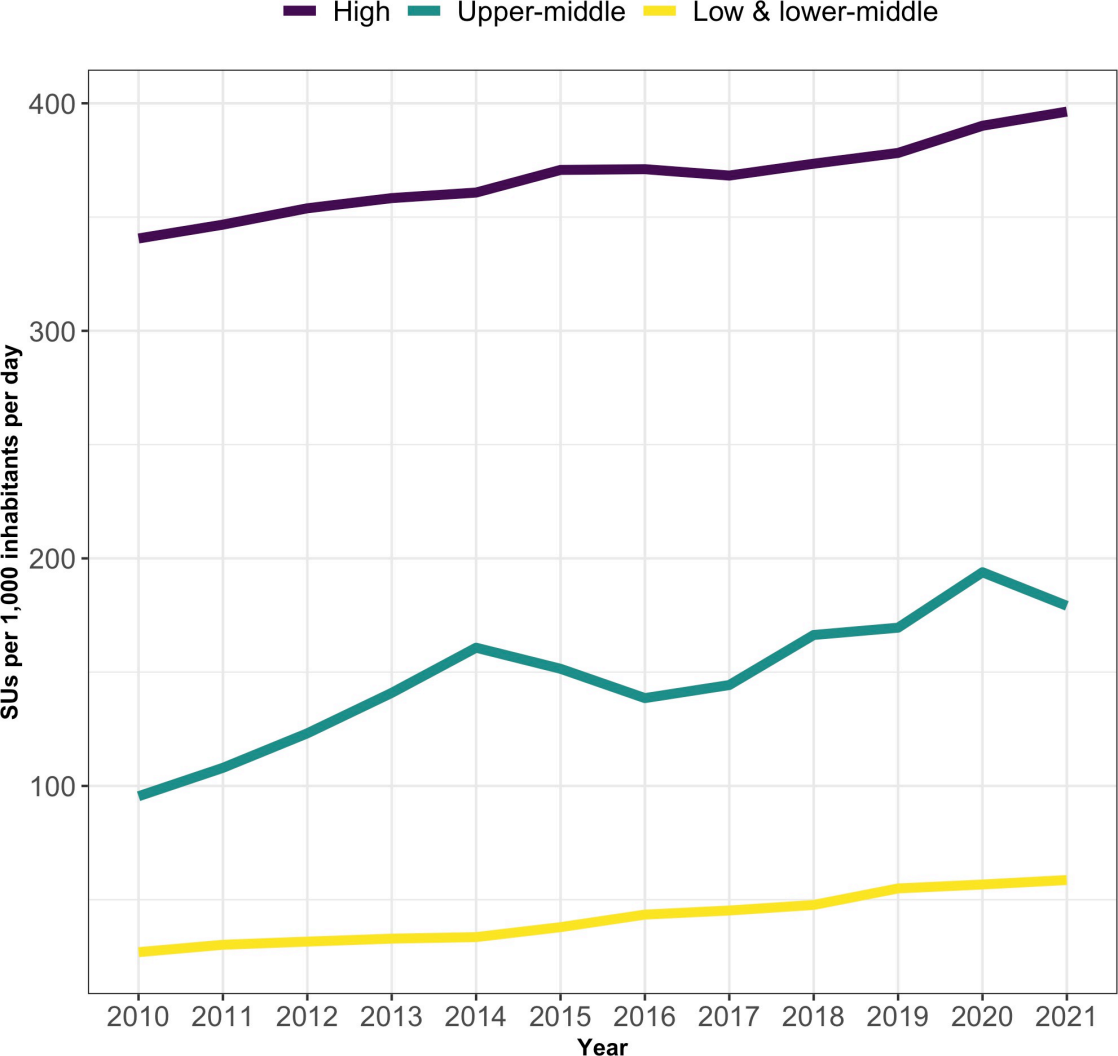
Median consumption rate of all antihypertensive medications from 2010–2021 in SUs, by country income class.

The global median consumption rate of all antihypertensive medications per 1,000 inhabitants per day increased from 184.78 SUs in 2010 to 325.6 SUs in 2021. In 2021, 47% of the global SU volume of antihypertensives was consumed by HICs, with 34% and 18% consumed by UMICs and LMICs, respectively. For all country income classes (LMIC, UMIC, and HIC), consumption rates consistently increased from 2010 to 2021 (Fig 2). HICs had consistently higher rates of consumption than UMICs, which in turn had consistently higher rates of consumption than LMICs for the entire study period (S2 Table). For LMICs, the median consumption rate in SUs increased from 26.9 SUs [IQR, 24.8] in 2010 to 58.6 SUs [IQR, 40.2] in 2021. For UMICs, the median consumption rate increased from 95.5 SUs [IQR, 112.9] to 179.2 SUs [IQR, 294.1] in 2021. For HICs, the median consumption rate increased from 340.6 SUs [IQR, 123.5] to 396.4 SUs [IQR, 178.2] in 2021. Median consumption rates in HICs in 2021 were more than four times the median consumption rate of LMICs. However, UMICs experienced the highest growth rate between 2010 and 2021. The consumption rate trends from 2010 to 2021 significantly differed between LMICS, UMICs, and HICs [Wald chi-square test, HIC and LMIC: chi2 = 155.74; p value < .001, HIC and UMIC: chi2 = 52.67; p value < .001, UMIC and LMIC: chi2 = 15.80; p value < .001]. Results for 2019 are reported in S1 Fig.

**Fig 3:**
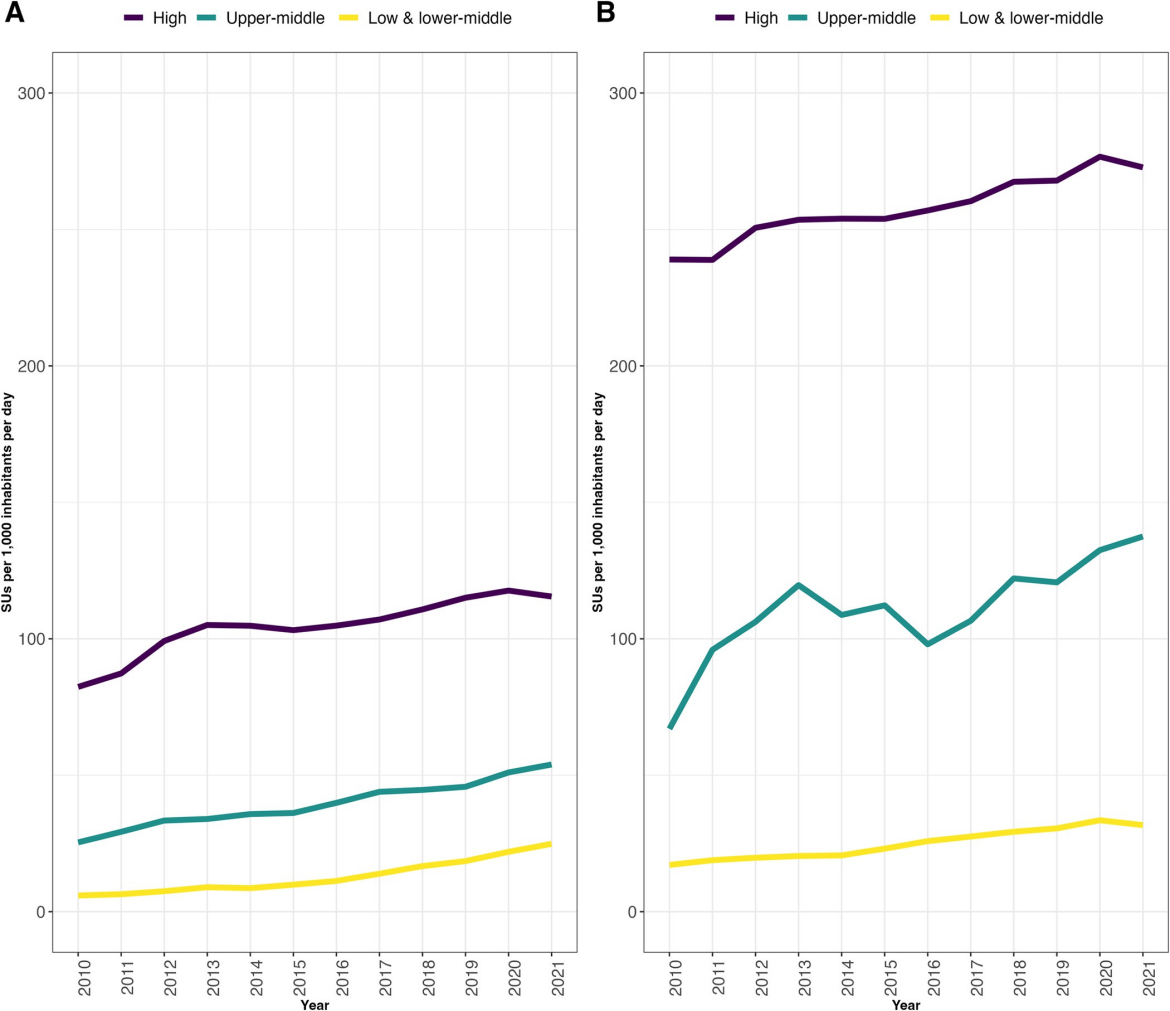
Median consumption rate for combination vs non-combination medications from 2010–2021 by country income class.

The median consumption rate of combination antihypertensive medications increased from 2010 to 2021 for HICs, UMICs, and LMICs (Fig 3A). HICs had the highest median consumption rates consistently across the study period, followed by UMICs and LMICs (S3 Table). LMIC median consumption rates nearly quadrupled between 2010 and 2021 (5.9 to 24.9), while UMICs approximately doubled (25.4 to 53.9). Echoing the trend for median consumption rates of all medications, HICs experienced the lowest percentage increase of all country income classes.

The median consumption rate of non-combination antihypertensive medications also increased from 2010 to 2021 for HICs, UMICs, and LMICs (Fig 3B). As with combination medications, HICs had the highest median consumption rate consistently across the study period, followed by UMICs and LMICs (S4 Table).

Across the entire study period of 2010 to 2021, the median consumption rates of combination medications were lower than those of non-combination medications for all country income classes (Fig 3). However, consumption rates of combination medications for all country classes did increase through the study period, though not enough to overtake consumption rates for non-combination medications. For example, in LMICs, median consumption rates of combination medications increased from 5.9 SUs to 24.8 SUs, while non-combination medications in the same time period increased from 17.2 SUs to 31.7 SUs.

**Fig 4:**
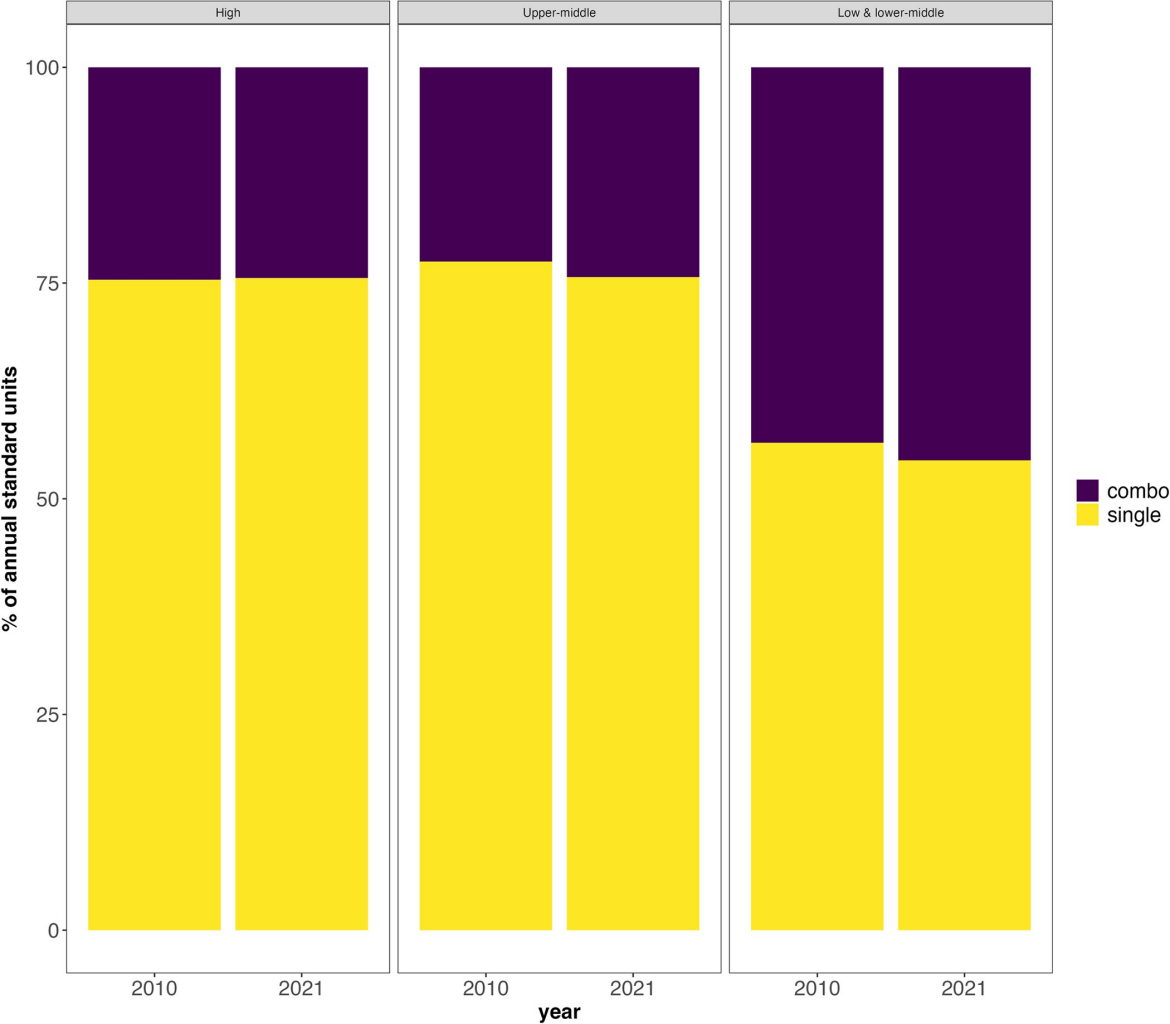
Consumption of combination vs non-combination drugs in 2010 and 2021 by percentage of annual SUs.

Based on SU volume of all antihypertensive medications in 2010 and 2021, non-combination medications were the most consumed compared to combination medications in LMICs, UMICs, and HICs (Fig 4). However, LMICs consumed a much higher percentage of combination drugs (45.5%) than UMICs (24.3%) and HICs (24.4%) in 2021. Between 2010 and 2021, both LMICs and UMICs slightly increased the proportion of combination drugs (43.5% to 45.5% and 22.5% to 24.3%, respectively). By contrast, the proportion of combination antihypertensive drugs consumed in HICs remained unchanged over the study period (24.6 to 24.4). Results for 2019 are reported in S2 Fig.

**Fig 5:**
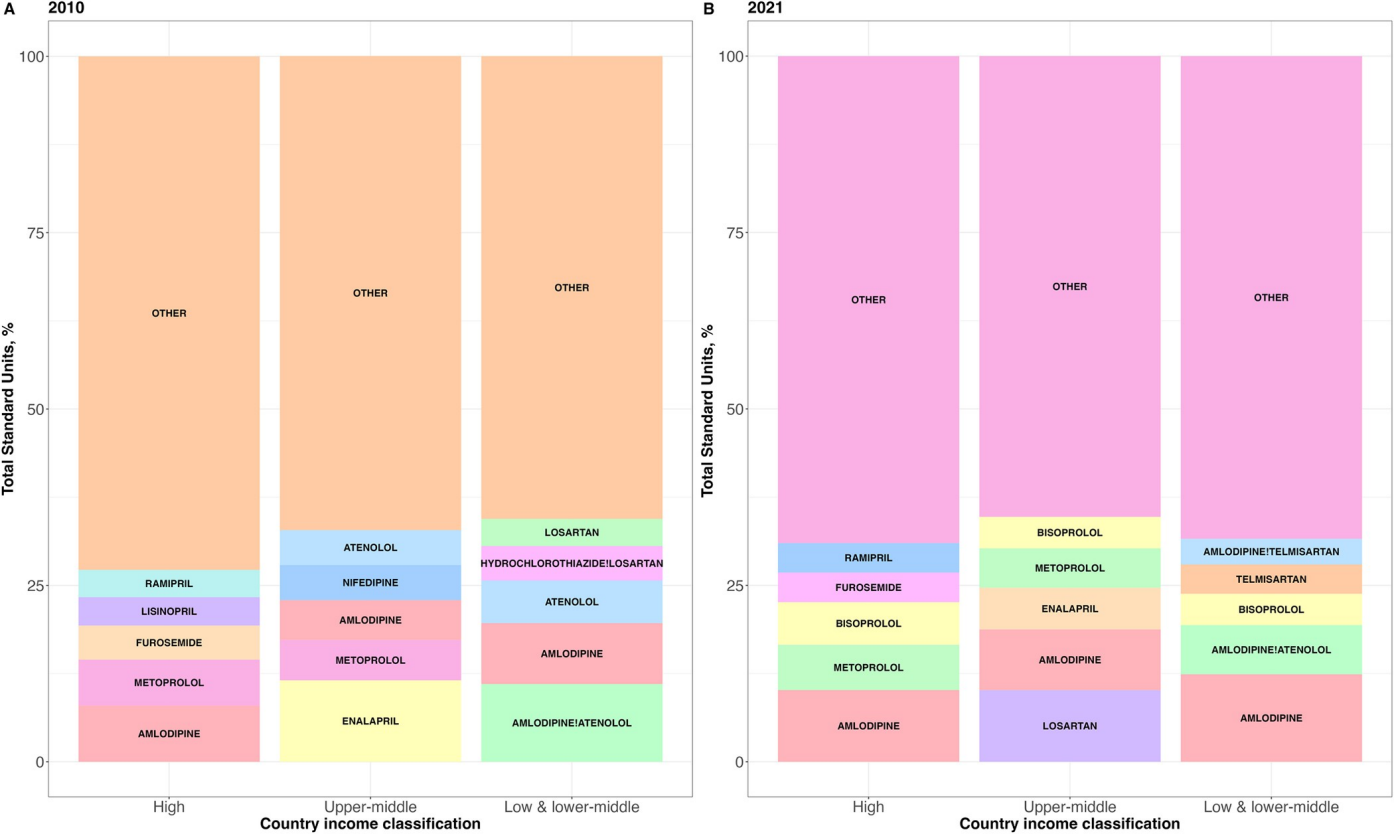
Top 5 most sold antihypertensive drugs in 2010 and 2021 in percentage of annual SUs by country income class. Top 5 drugs in the “other” category in 2010 include: high-income—BISOPROLOL(3.3%), ATENOLOL(3.2%), ENALAPRIL(2.7%), CARVEDILOL(2.7%), HYDROCHLOROTHIAZIDE!VALSARTAN(2.6%); upper-middle–HYDROCHLOROTHIAZIDE(4.3%), CAPTOPRIL(4.2%), INDAPAMIDE(3.2%), FUROSEMIDE(3.1%), LOSARTAN(3%); low & lower-middle–METOPROLOL(3.7%), NIFEDIPINE(3.3%), CAPTOPRIL(3.1%), ENALAPRIL(3.1%), PROPRANOLOL(3.1%). Top 5 drugs in the “other” category in 2021 include: high-income–LOSARTAN(3.7%), LISINOPRIL(3.7%), CARVEDILOL(3%), CANDESARTAN CILEXETIL(2.6%), HYDROCHLOROTHIAZIDE(2.3%); upper-middle–NIFEDIPINE(4.1%), HYDROCHLOROTHIAZIDE(4.0%), ATENOLOL(2.6%), FUROSEMIDE(2.6%), SPIRONOLACTONE(2.4%); low & lower-middle–LOSARTAN(3.5%), METOPROLOL(3.3%), HYDROCHLOROTHIAZIDE!TELMISARTAN(3.3%), PROPRANOLOL(2.4%), HYDROCHLOROTHIAZIDE!LOSARTAN(2.4%).

Combination medications were among the top five most sold medications in both 2010 and 2021 for LMICs only (Fig 5). UMICs and HICs did not have any combination medications among their top 5 medications consumed by volume in both 2010 and 2021. Within the top 10 medications consumed by volume, LMICs had four combination medications, two of which are WHO EML listed (amlodipine/telmisartan and hydrochlorothiazide/telmisartan). By contrast, UMICs and HICs only had one combination medication listed per country class in their top 10 drugs (Apocynum venetum and hydrochlorothiazide/losartan, respectively).

## Discussion

HICs had the highest antihypertensive consumption rate, four times the median rate of LMICs in 2021. However, UMICs and LMICs consume a higher percentage of combination antihypertensive medications, a potentially more effective and clinically recommended first-line therapy for patients with hypertension. LMICs consumed a higher percentage of combination medications out of total antihypertensive medications than UMICs and HICs. While HICs had higher consumption rates of antihypertensive medications, they consumed a lower proportion of combination medications relative to non-combination antihypertensive drugs. Therefore, even when clinical consensus broadly exists and recommends single-pill combination antihypertensive medication for hypertension control, there is inconsistent global uptake of such recommendations.

Combination antihypertensive medications commonly combine two drugs from different mechanistic categories to maximize clinical benefit—using two drugs can not only target hypertension from two different pathophysiological pathways but also reduce the possibilities of adverse side effects from using a higher dose of one class on its own. Other diseases have also used combination medications to simplify treatment. Combination medications have previously been recommended as first-line treatment in other diseases like tuberculosis and HIV. Like hypertension, tuberculosis and HIV both required consistent dosing of multiple pills a day, resulting in low patient adherence and logistical complications due to requiring sourcing of each individual pill. Combination pills for HIV revolutionized implementation of therapy. Instead of requiring multiple pills multiple times a day, and requiring providers to individually prescribe each pill, a combination pill for HIV not only simplified the task required of prescribers but also simplified treatment regimens for patients [18]. Combination pills for HIV have been shown to increase patient adherence, increasing the likelihood of sustained adherence and HIV viral load control over time [19].

Many of the same advantages that combination medications held for HIV are true for hypertension treatment. Hypertension is a disease that requires frequent medication dosing from different classes for most patients. Combination medications not only reduce treatment complexity but help improve patient adherence. The 2021 WHO guideline for the pharmacological treatment of hypertension in adults recommended combination therapy (single-pill combination) as initial treatment [13]. The 2018 European Society of Cardiology/European Society of Hypertension released new guidelines that emphasize medical professionals to initiate treatment with combination therapy because most patients will not reach target blood pressure measurements with monotherapies [20]. Similarly, the 2017 American College of Cardiology/American Heart Association guidelines for the management of high blood pressure recommended combination therapy to increase adherence to the medication [21]. Additionally, there are significant supply-chain advantages to combination drugs where supply chain costs could be reduced due to simplifications in production, storage and dispensing [18].

However, actual consumption patterns for combination antihypertensive medications are not well understood. Previous studies have analysed consumption of antihypertensive drugs, but have not examined combination hypertensive medication consumption relative to non-combination antihypertensive drugs [22–26]. With combination antihypertensive medications now a first-line recommended hypertension medication by multiple clinical body guidelines, understanding consumption of combination medications relative to non-combination medications is crucial to devising policy responses for efficient use of existing resources. To our knowledge, this is the first study to track consumption rates globally for all combination and non-combination antihypertensive drugs over a decade, demonstrating longitudinal patterns of consumption as different clinical standards were established.

Several limitations should be considered when interpreting these findings. We assumed sales data to approximate consumption. Some pharmaceuticals that are purchased may not be used due to a range of distribution and healthcare related factors. Additionally, country-specific protocols and disease severity classifications may influence drug purchases. This study also used Standard Units (SUs) to standardize dosing between medications, which is a unit defined by IQVIA to be one dose of a medication no matter what form (i.e., pill, injection, powder). This was used to standardize dosing of different classes of medications which all have different strengths and dosing schedules according to clinical guidelines. This may affect estimates of consumption, as it does not consider how dosing from one medication may change between medication classes. However, previous studies have also used SUs in studies concerning medication consumption because it allows for standardization and calculation of overall consumption trends in a uniform way [15, 16, 27]. We also included all types of antihypertension medications in our analysis to have a robust and well represented picture of consumption. Regional preferences still exist in prescribing patterns regarding different types of medications and including all types of medication that could be used for hypertension allowed us to capture as much global consumption as possible. However, some medications used for blood pressure also have additional indications (i.e., diuretics used for blood pressure control are also used for treatment of heart failure) and we were unable to exclude consumption for these additional indications, although such use is considerably small and may not influence these patterns substantially. Lastly, we did not have data on drug costs and therefore could not assess the impact of price differences between combination and non-combination antihypertensives.

Our findings suggest that existing policies encouraging consumption of combination medications (clinical guidelines, global purchasing agreements) have worked to encourage consumption of antihypertensives in LMICs but not in UMICs and HICs. Given the efficiency and efficacy benefits of combination medications in reducing hypertension, UMICs and HICs have significant room to reduce costs and improve rates of hypertension control by increasing the use of combination antihypertensive drugs relative to non-combination drugs. In LMICs, however, rates of all antihypertensive consumption were and remained significantly below UMICs and HICs, for the duration of the study period.

## Supporting information

S1 Table Retail sector antihypertensives market share for countries with missing hospital sector data. (DOCX)

S2 Table Consumption rates for all antihypertensive drugs, SUs per 1000 inhabitants per day. (DOCX)

S3 Table Consumption rates for combination antihypertensive drugs, SUs per 1000 inhabitants per day. (DOCX)

S4 Table Consumption rates for non-combination antihypertensive drugs, SUs per 1000 inhabitants per day. (DOCX)

S1 Fig SU consumption rate by country for 2019 in SUs per 1000 inhabitants per day. (TIF)

S2 Fig Consumption of combination vs non-combination drugs in 2010 and 2019 by percentage of annual SUs. (TIF)
