# Supplementary material for: Global consumption patterns of combination hypertension medication: An analysis of pharmaceutical sales data from 2010–2021
Source: PLOS Glob Public Health. 2024 Sep 6;4(9):e0003698. doi: 10.1371/journal.pgph.0003698 (PMC11379295; doi:10.1371/journal.pgph.0003698)
Supplement: S1 Table — (DOCX) [file pgph.0003698.s001.docx]

**S1 Table: Retail sector antihypertensives market share for countries with missing hospital sector data**

| **Country** | **Retail sector market share, %** |
| --- | --- |
|  |  |
| ALGERIA | 80% |
| ARGENTINA | 73% |
| BANGLADESH | 85% |
| BOSNIA | 75% |
| CENTRAL AMERICA | 77% |
| CHILE | 71% |
| COLOMBIA | 71% |
| DOMINICAN REPUBLIC | 78% |
| EGYPT | 75% |
| ESTONIA | 88% |
| FRENCH WEST AFRICA | 86% |
| GREECE | 60% |
| HONG KONG | 91% |
| INDONESIA | 60% |
| JORDAN | 71% |
| KUWAIT | 35% |
| LEBANON | 77% |
| LUXEMBOURG | 97% |
| MOROCCO | 88% |
| PAKISTAN | 85% |
| PERU | 67% |
| SERBIA | 76% |
| SLOVENIA | 73% |
| SRI LANKA | 58% |
| UAE | 45% |
| URUGUAY | 69% |
| VENEZUELA | 78% |
