# Supplementary material for: Global consumption patterns of combination hypertension medication: An analysis of pharmaceutical sales data from 2010–2021
Source: PLOS Glob Public Health. 2024 Sep 6;4(9):e0003698. doi: 10.1371/journal.pgph.0003698 (PMC11379295; doi:10.1371/journal.pgph.0003698)
Supplement: S2 Table — (DOCX) [file pgph.0003698.s002.docx]

**S2 Table: Consumption rates for all antihypertensive drugs, SUs per 1000 inhabitants per day**

| **Income classification** | **Year** | **Median consumption rate** | **Interquartile range(IQR)** |
| --- | --- | --- | --- |
| High | 2010 | 340.60 | 123.55 |
| High | 2011 | 346.67 | 105.95 |
| High | 2012 | 353.86 | 107.99 |
| High | 2013 | 358.34 | 137.61 |
| High | 2014 | 360.77 | 138.46 |
| High | 2015 | 370.72 | 131.47 |
| High | 2016 | 371.04 | 143.87 |
| High | 2017 | 368.24 | 152.90 |
| High | 2018 | 373.46 | 161.09 |
| High | 2019 | 378.19 | 172.74 |
| High | 2020 | 390.13 | 179.29 |
| High | 2021 | 396.35 | 178.15 |
| Upper-middle | 2010 | 95.46 | 112.92 |
| Upper-middle | 2011 | 107.95 | 134.81 |
| Upper-middle | 2012 | 123.12 | 159.57 |
| Upper-middle | 2013 | 140.90 | 175.18 |
| Upper-middle | 2014 | 160.75 | 188.11 |
| Upper-middle | 2015 | 151.49 | 197.27 |
| Upper-middle | 2016 | 138.59 | 211.67 |
| Upper-middle | 2017 | 144.34 | 245.36 |
| Upper-middle | 2018 | 166.33 | 261.32 |
| Upper-middle | 2019 | 169.54 | 272.34 |
| Upper-middle | 2020 | 193.91 | 306.22 |
| Upper-middle | 2021 | 179.15 | 294.13 |
| Low & lower-middle | 2010 | 26.95 | 24.89 |
| Low & lower-middle | 2011 | 30.19 | 30.12 |
| Low & lower-middle | 2012 | 31.58 | 32.71 |
| Low & lower-middle | 2013 | 32.88 | 30.50 |
| Low & lower-middle | 2014 | 33.56 | 35.75 |
| Low & lower-middle | 2015 | 37.93 | 34.71 |
| Low & lower-middle | 2016 | 43.45 | 42.05 |
| Low & lower-middle | 2017 | 45.24 | 31.85 |
| Low & lower-middle | 2018 | 47.70 | 37.51 |
| Low & lower-middle | 2019 | 54.94 | 43.51 |
| Low & lower-middle | 2020 | 56.68 | 42.80 |
| Low & lower-middle | 2021 | 58.64 | 40.16 |
