# Supplementary material for: Global consumption patterns of combination hypertension medication: An analysis of pharmaceutical sales data from 2010–2021
Source: PLOS Glob Public Health. 2024 Sep 6;4(9):e0003698. doi: 10.1371/journal.pgph.0003698 (PMC11379295; doi:10.1371/journal.pgph.0003698)
Supplement: S3 Table — (DOCX) [file pgph.0003698.s003.docx]

**S3 Table: Consumption rates for combination antihypertensive drugs, SUs per 1000 inhabitants per day**

| **Income classification** | **Year** | **Median consumption rate** | **Interquartile range(IQR)** |
| --- | --- | --- | --- |
| High | 2010 | 82.37 | 99.01 |
| High | 2011 | 87.30 | 95.55 |
| High | 2012 | 99.19 | 97.30 |
| High | 2013 | 105.06 | 93.02 |
| High | 2014 | 104.78 | 96.85 |
| High | 2015 | 103.17 | 106.54 |
| High | 2016 | 104.82 | 109.86 |
| High | 2017 | 107.07 | 116.83 |
| High | 2018 | 110.77 | 118.25 |
| High | 2019 | 115.05 | 117.62 |
| High | 2020 | 117.67 | 121.98 |
| High | 2021 | 115.49 | 122.26 |
| Upper-middle | 2010 | 25.39 | 25.14 |
| Upper-middle | 2011 | 29.27 | 27.27 |
| Upper-middle | 2012 | 33.40 | 23.78 |
| Upper-middle | 2013 | 33.98 | 28.24 |
| Upper-middle | 2014 | 35.76 | 32.21 |
| Upper-middle | 2015 | 36.16 | 38.55 |
| Upper-middle | 2016 | 39.88 | 47.10 |
| Upper-middle | 2017 | 43.93 | 60.94 |
| Upper-middle | 2018 | 44.63 | 69.54 |
| Upper-middle | 2019 | 45.76 | 82.29 |
| Upper-middle | 2020 | 51.03 | 97.53 |
| Upper-middle | 2021 | 53.93 | 95.53 |
| Low & lower-middle | 2010 | 5.94 | 12.65 |
| Low & lower-middle | 2011 | 6.41 | 14.22 |
| Low & lower-middle | 2012 | 7.50 | 14.98 |
| Low & lower-middle | 2013 | 8.96 | 16.24 |
| Low & lower-middle | 2014 | 8.62 | 17.43 |
| Low & lower-middle | 2015 | 9.90 | 19.77 |
| Low & lower-middle | 2016 | 11.27 | 21.63 |
| Low & lower-middle | 2017 | 13.92 | 22.54 |
| Low & lower-middle | 2018 | 16.71 | 23.90 |
| Low & lower-middle | 2019 | 18.54 | 26.17 |
| Low & lower-middle | 2020 | 21.94 | 29.01 |
| Low & lower-middle | 2021 | 24.87 | 29.15 |
