# Supplementary material for: Global consumption patterns of combination hypertension medication: An analysis of pharmaceutical sales data from 2010–2021
Source: PLOS Glob Public Health. 2024 Sep 6;4(9):e0003698. doi: 10.1371/journal.pgph.0003698 (PMC11379295; doi:10.1371/journal.pgph.0003698)
Supplement: S4 Table — (DOCX) [file pgph.0003698.s004.docx]

**S4 Table: Consumption rates for non-combination antihypertensive drugs, SUs per 1000 inhabitants per day**

| **Income classification** | **Year** | **Median consumption rate** | **Interquartile range(IQR)** |
| --- | --- | --- | --- |
| High | 2010 | 238.98 | 107.27 |
| High | 2011 | 238.85 | 106.06 |
| High | 2012 | 250.60 | 95.94 |
| High | 2013 | 253.55 | 91.38 |
| High | 2014 | 253.95 | 92.70 |
| High | 2015 | 253.88 | 102.18 |
| High | 2016 | 256.95 | 111.69 |
| High | 2017 | 260.36 | 109.75 |
| High | 2018 | 267.46 | 114.99 |
| High | 2019 | 267.91 | 123.41 |
| High | 2020 | 276.65 | 105.02 |
| High | 2021 | 272.75 | 107.83 |
| Upper-middle | 2010 | 67.00 | 88.41 |
| Upper-middle | 2011 | 95.97 | 109.24 |
| Upper-middle | 2012 | 106.22 | 135.28 |
| Upper-middle | 2013 | 119.68 | 159.32 |
| Upper-middle | 2014 | 108.75 | 168.41 |
| Upper-middle | 2015 | 112.26 | 184.52 |
| Upper-middle | 2016 | 98.00 | 195.80 |
| Upper-middle | 2017 | 106.60 | 229.82 |
| Upper-middle | 2018 | 122.13 | 240.75 |
| Upper-middle | 2019 | 120.67 | 245.99 |
| Upper-middle | 2020 | 132.49 | 266.42 |
| Upper-middle | 2021 | 137.49 | 268.83 |
| Low & lower-middle | 2010 | 17.15 | 17.17 |
| Low & lower-middle | 2011 | 18.87 | 25.70 |
| Low & lower-middle | 2012 | 19.77 | 27.93 |
| Low & lower-middle | 2013 | 20.42 | 26.65 |
| Low & lower-middle | 2014 | 20.64 | 25.76 |
| Low & lower-middle | 2015 | 23.10 | 23.21 |
| Low & lower-middle | 2016 | 25.84 | 26.18 |
| Low & lower-middle | 2017 | 27.54 | 24.15 |
| Low & lower-middle | 2018 | 29.28 | 24.86 |
| Low & lower-middle | 2019 | 30.54 | 30.35 |
| Low & lower-middle | 2020 | 33.50 | 33.56 |
| Low & lower-middle | 2021 | 31.71 | 40.09 |
