# Supplementary figures and images for: Global consumption patterns of combination hypertension medication: An analysis of pharmaceutical sales data from 2010–2021
Source: PLOS Glob Public Health. 2024 Sep 6;4(9):e0003698. doi: 10.1371/journal.pgph.0003698 (PMC11379295; doi:10.1371/journal.pgph.0003698)

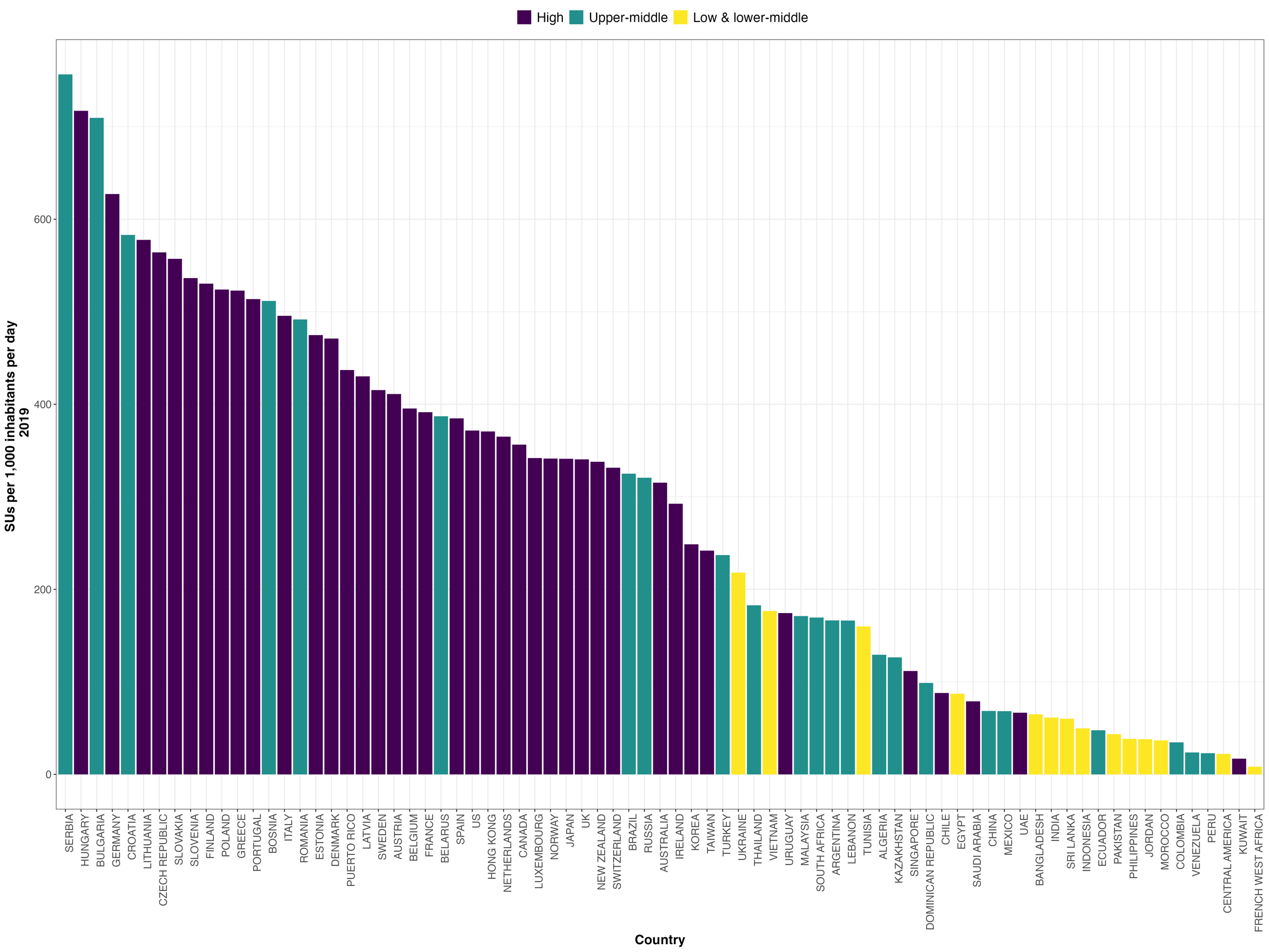

Supplement: S1 Fig — (TIF) [file pgph.0003698.s005.tif]

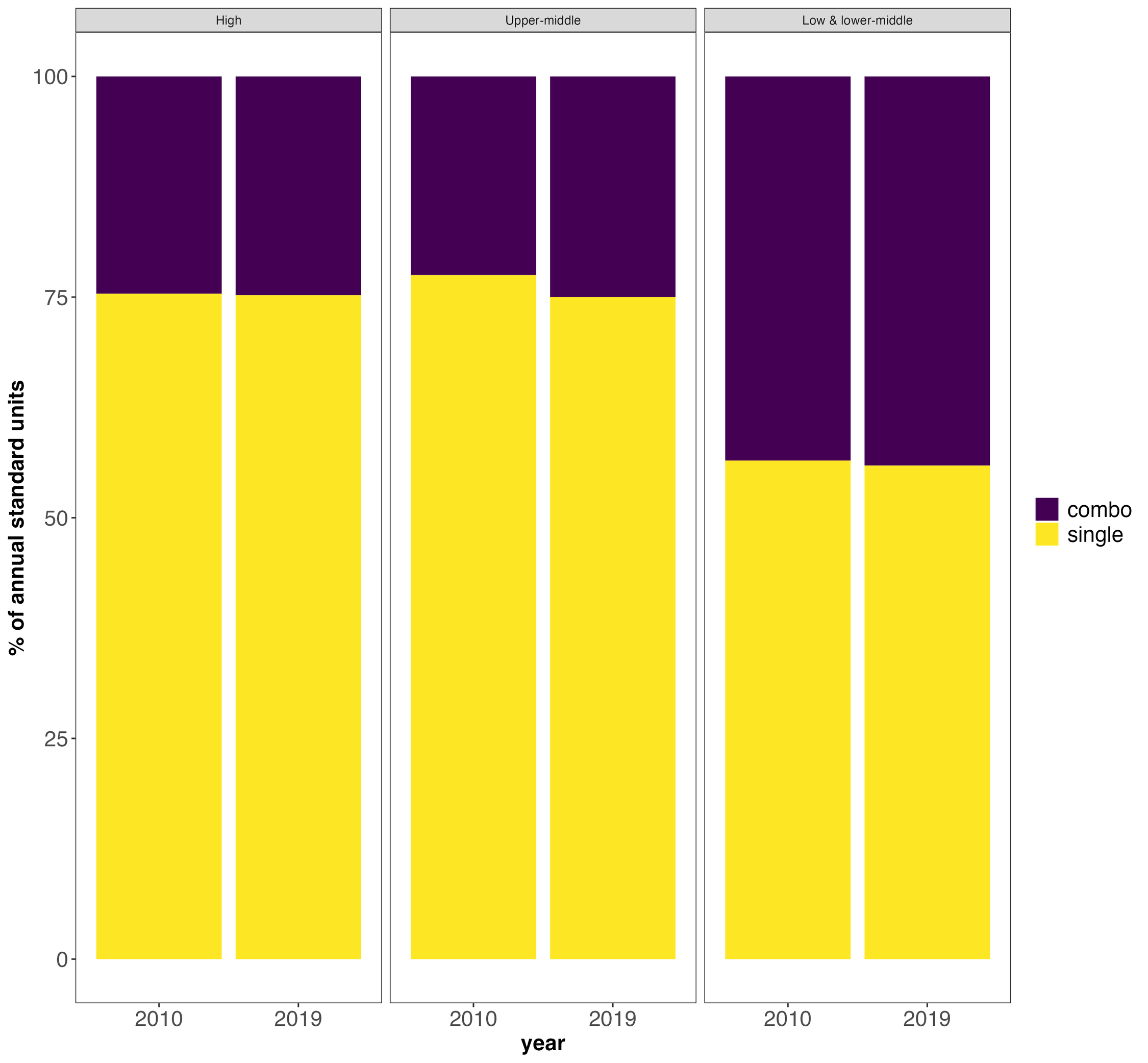

Supplement: S2 Fig — (TIF) [file pgph.0003698.s006.tif]
